# Ensuring Patient Safety in Emergency Peripheral Ultrasound-Guided Nerve Blocks: An Evaluation of a Quality Improvement/Patient Safety Initiative

**DOI:** 10.51894/001c.7402

**Published:** 2019-03-04

**Authors:** Daniel J. Wahl, Andrew J. Butki, Nikolai Butki, Samuel J. Wisniewski

**Affiliations:** 1 Emergency Medicine McLaren Oakland; 2 College of Osteopathic Medicine Michigan State University, East Lansing, MI

**Keywords:** patient safety, emergency medicine, peripheral nerve-block procedures, ultrasound

## Abstract

**CONTEXT:**

During the past two decades, bedside ultrasound has revolutionized the practice of emergency medicine. Physicians are now expected to be competent in utilizing ultrasound skills, for patients presenting with conditions ranging from trauma to skin evaluations. The overall purpose of this quality improvement/patient safety (QIPS) project was to evaluate the effectiveness of a pair of five-hour, hands-on didactic/training sessions, aimed at preparing a sample of emergency medicine physicians, residents and medical students to perform peripheral ultrasound-guided nerve blocks.

**METHODS:**

The study location was set in a community-based emergency medicine program in Pontiac, Michigan. Data was collected from N = 54 emergency medicine residents, physicians and medical students. Data was collected from two training sessions in November 2017 and January 2018. The training consisted of a 12-question pre-test, followed by five hours of hands on & didactic training, with a subsequent post-test containing the same questions.

**RESULTS:**

The authors compiled the data from both training sessions and found that the participants had an average correct percentage of 5.52 of 12 (46%) on the pre-test. After attending the training session, participants had an overall correct percentage of 9.24 of 12 (77%) on the post-test. This pre-to post-training increase of the mean scores was statistically significant, t (53) = -10.76 (p < 0.01), with an effect size (Cohen’s d) of 1.82. Post hoc power calculations utilizing the d = 1.82 effect size revealed statistical power (1- *β*) of 100%.

**CONCLUSIONS:**

The results of this QIPS evaluation project suggest that emergency physicians, residents and medical students may achieve an improved understanding of key ultrasound-guided nerve block material after a single five-hour session of hands-on training and didactics. Going forward, additional studies employing larger sample sizes that allow for outcome stratification by group (emergency physicians, residents, or medical students) along with relevant demographic variables (age, years in practice, etc.) in similar settings are needed to further verify these findings.

## INTRODUCTION

During the past two decades, bedside ultrasound has revolutionized the practice of emergency medicine (EM). The next generation of emergency physicians are expected to be competent in utilizing ultrasound (US) skills for patients presenting with conditions ranging from trauma to skin evaluations.^1^ A rapidly evolving use of bedside ultrasound in EM is combining fine motor skills with knowledge of peripheral nervous system anatomy and physiology to perform US-guided nerve blocks.^2^

The history of US-guided regional anesthesia has quickly evolved over the last 25 years.^3^ In 1989, Ting and Sivagnanaratnam described using ultrasonography to localize a needle while performing an axillary nerve block.^4^ They reported no patient complications, due to visualizing the needle and surrounding anatomy at all times.^4^ In 1994, Kapral et al. demonstrated the benefits of US for supraclavicular blocks.^5^ Subsequent studies have demonstrated that the use of US guidance allowed for smaller amounts of local anesthetic to produce an effective nerve block.^6^

As US technology improved access at the bedside in emergency department (ED) settings, a team of Toronto physicians in 2003 was able to demonstrate adequate localization of patients’ brachial plexuses with high-quality images.^7^ Since then, the use of bedside US has revolutionized EM, particularly in regional anesthesia. In 2006, Blaivas and Lyon described four cases of shoulder dislocations, in which regional anesthesia was successful after performing US-guided interscalene brachial plexus blocks.^8^ In 2010, Chandra, et al. published a paper describing the history and patient benefits of US-guided nerve blocks in the ED.^9^

The benefits of performing peripheral US-guided nerve blocks in ED settings are numerous. They range from joint dislocation reduction, wound care, fracture reduction, decreased use of procedural sedation and lower amounts of opioids required to reduce pain.^10^ The risk of iatrogenic injury or complications from US-guided nerve blocks has been shown to be lower than when performed blindly.^11^ However, there is still potential for unintended intravascular injections of local anesthetic, local anesthetic systemic toxicity, intraneural injections, accidental vascular punctures, hematoma formation, pneumothorax, allergy to local anesthetic, and infection.^12^ However, multiple earlier studies have demonstrated that providers can perform US-guided nerve blocks successfully in both pediatric and adult patients in the ED setting.^13–22^

### Project Purpose

The purpose of this quality improvement/patient safety (QIPS) project was to evaluate the effectiveness of a single five-hour, hands-on didactic/training session at preparing EM physicians, residents and medical students to perform peripheral US-guided nerve blocks. During training sessions, participants were also taught how to recognize and treat potential complications from US-guided nerve blocks. Participants’ understanding of training session content was evaluated utilizing pre- and post-session test scores. The authors’ goal was to demonstrate a statistically significant improvement between pre- and post-test scores.

## METHODS

IRB exemption was obtained from McLaren IRB prior to conducting the US training. Participants were EM physicians, residents and a small number of medical students. Learners were administered a knowledge quiz comprised of 12 questions for the pre-test without knowing the correct responses (Appendix A). The questions were created by the first and second authors of this paper (DJW and AJB). Data for both the pre- and post-tests utilized Kahoot “Learning Games|Make Learning Awesome!” as the digital platform for trainees to submit their answers in real-time, via their personal cell phones, tablets or computers.^23^

The didactic and motor skills training consisted of a five-hour training session. The training began with a one-hour US didactic presentation, which covered patient safety topics associated with providing peripheral US-guided nerve blocks. Specific topics included: a) dosing for regional anesthesia, b) appropriate monitoring to ensure patient safety, c) intralipid antidote for local toxicity and d) duration of action of local anesthetics. Participants were also taught the anatomy of specific nerves and their surrounding structures. That knowledge was then applied in live-session training, as participants gained the key technical skills needed to provide US-guided nerve blocks.

The specific nerve blocks taught included the Median n., Ulnar n., Radial n., Femoral n., Popliteal n. and Tibial n. These nerve blocks were taught by the first two authors (DJW and AJB), who are trained in US-guided nerve blocks (New York School of Regional Anesthesia and Emergency Ultrasound Fellowship, respectively).^24^ The nerve blocks that were taught during this course were selected based on level of difficulty, usefulness in routine EM care and relative safety profile.^25–27^

Learners’ motor skills were developed during breakout sessions, during which participants identified the six previously listed nerves using US on participant colleagues. Participants also used a Blue Phantom^TM^ nerve block task trainer to acquire the motor skills of needle-nerve localization and anesthetic injection.^28^

Following this didactic and motor skills training, participants were asked to take the same 12-item test as a post-test. Training sessions were performed on two separate days to maximize participants. The trainings took place in November 2017 and January 2018, with a total of 54 participants. The same trainers (DJW and AJB) taught both sessions, for consistency of material delivery. Results were not analyzed until after the second training session. The results of participants were only analyzed for those individuals who had completed both the pre- and post-tests and attended the entirety of the didactic and motor skills training sessions.

### Statistical Analysis

Pre- and post-test scores were first compared on a base descriptive level for each day of training (e.g., one in November 2017, and one in January 2018). Post hoc two-tailed power calculations assuming a moderate effect size (0.5) and an alpha of 0.05 were also performed. After verifying distributional assumptions, a series of Wilcoxon Matched Paired t-Tests were also performed comparing pre- and post- mean test scores for all participants over the two days of training. All statistical analyses were performed by the fourth author (SJW) utilizing SPSS Version 25 analytic software.^29^

## RESULTS

Thirty-six participants were enrolled the first training day in November 2017. The participants scored an average overall correct response of 4.89 out of 12 (41%) on the pre-test. Subsequently, the participants scored an average overall correct response of 8.78 out of 12 (73%) on the post-test. Eighteen participants were enrolled on the second training day, in January 2018. The participants scored an average overall correct response of 6.78 out of 12 (57%) on the pre-test. The participants subsequently scored an average overall correct response of 10.17 out of 12 (85%) on the post-test.

The combined results of the 54 participants scored an average overall correct response of 5.52 out of 12 (46%) on the pre-test. The combined participants scored an average overall correct response of 9.24 out of 12 (77%) on the post-test. This pre-to-post increase in mean scores were statistically significant, *t* (53) = -10.76 (p < 0.001), with an effect size (Cohen’s *d*) of 1.82. Post hoc power calculations utilizing the *d* = 1.82 effect size revealed statistical power (1- *β*) of 100%.

## DISCUSSION

Complications from peripheral nerve blocks are rare, but can be potentially catastrophic (e.g., systemic local anesthetic toxicity).^30^ As US-guided regional anesthesia continues to be increasingly utilized in the ED, the authors aimed to assess how effectively key concepts about patient safety were being taught. The group composition on the two training days of the study did not vary significantly, with similar pre- and post-test score improvements obtained from both groups.

The authors’ goal of obtaining a statistically significant overall improvement between the pre- and post-test scores was consistently realized. The combined results of participants’ correct pre-test answers was a mere 46% (n = 54) for the series of safety questions about local anesthetic dosage, concentration, nerve block technique, etc. (Appendix A). Following the training sessions, the combined participants scored an average overall correct response of 77% (n = 54) on post-tests. This suggests that our training protocol may have been effective at introducing important patient safety considerations for peripheral US-guided nerve blocks to participants.

### Limitations

After analyzing our results and study design, we have identified several project limitations. First, we collected limited demographic data about the participants. We had only asked participants for information concerning their current training status (i.e., attending physician, resident physician and medical student). In future studies, demographic variables such as years in practice, number of years utilizing US technologies in EM settings and age of participants could be helpful for more detailed sub-group analysis. Additionally, the size of our community-based convenience sample was small. Although this project enrolled the majority of EM physicians in our Pontiac, Michigan institution, a larger sample size would be needed to perform more granular subgroup analyses. Furthermore, the pre- and post-session knowledge tests utilized during this study had not been previously validated. The questions were designed to target what the first two authors concluded to be the most important safety aspects of performing US-guided nerve blocks. Future training studies could include validated exams to more fully analyze learner outcomes.

## CONCLUSIONS

Since ultrasound technology’s early adoption in the late 1980s, more powerful bedside machines are now readily available. This has allowed US-guided nerve blocks to become more common in today’s emergency medicine practice. As ultrasound technology has improved, so have the skills of those performing bedside US procedures. As a profession, we need to ensure that patient safety knowledge escalates at a similar rate of skill acquisition. These project results demonstrate the potential for success in teaching patient safety to EM physicians, residents and medical students to perform US-guided nerve blocks. In the future, similarly structured training protocols could be implemented when teaching emergency physicians to perform these valuable patient treatment skills with a bedside ultrasound.

The review of this manuscript was coordinated by SMRJ Chief Editor William Corser.

### Conflict of Interest

The authors declare no conflict of interest.

## Supplementary Material

Appendix A Ultrasound Nerve Block Pre and Post-Test Questions with Answers (correct answers in bold)
