# Supplementary material for: Ensuring Patient Safety in Emergency Peripheral Ultrasound-Guided Nerve Blocks: An Evaluation of a Quality Improvement/Patient Safety Initiative
Source: Spartan Med Res J. 2019 Mar 4;3(3):7402. doi: 10.51894/001c.7402 (PMC7746071; doi:10.51894/001c.7402)
Supplement: Appendix A Ultrasound Nerve Block Pre and Post-Test Questions with Answers (correct answers in bold) [file smrj_2019_3_3_7402_18397.docx]

**Appendix A**

**Ultrasound Nerve Block Pre and Post-Test Questions with Answers**

**(correct answers in bold)**

1. What is the maximum amount of lidocaine without epinephrine that can be safely administered into the soft tissue?

3.5 mg/kg

7 mg/kg

**4.5 mg/kg**

8 mg/kg

2. What is the maximum amount of lidocaine with epinephrine that can be safely administered into the soft tissue?

4 mg/kg

**7 mg/kg**

5 mg/kg

8 mg/kg

3. What is the concentration per mL of 1% lidocaine without epi?

12 mg/ml

15 mg/ml

20 mg/ml

**10 mg/ml**

4. What medication needs to be administered quickly via IV in the event of a toxic reaction to local anesthesia?

Glucagon

D50

**Intralipid**

Pyridoxine

5. For the majority of ultrasound guided nerve blocks, what needle approach is recommended to maximize safety?

Seldinger technique

**In-plane technique**

Out-of-plane technique

Oblique technique

6. Of the medications listed below, which one is the most cardio-toxic?

**Bupivacaine**

Lidocaine without epi

Ropivacaine

Lidocaine with epi

7. What is the earliest sign that lidocaine with epinephrine has entered the blood stream?

Increased salivary secretions

**Increased heart rate**

Increased respirations

Decreased hearing

8. A patient has a large laceration. Local infiltration of 1% lidocaine without epinephrine lasts roughly 45 minutes. How long (anesthesia time) does a typical block using the same lidocaine without epinephrine last?

45-60 min

**90-180 min**

180-260 min

30-60 min

9. What are the EKG findings in local anesthesia toxicity?

Narrow QRS, Bradycardia, Hypertension

Narrow QRS, Tachycardia, Hypertension

Wide QRS, Tachycardia, Hypotension

**Wide QRS, Bradycardia, Hypotension**

10. When using 0.5% Ropivacaine for a block, what is the expected onset time for the block to begin working?

**15-30 min**

10-15 min

5-10 min

Less than 5 min

11. What is recommended dose of Lipid Emulsion for a 70 kg lean body weight adult?

70 mL

**100 mL**

120 mL

150 mL

12. Once the needle is through the fascial plane, if you encounter resistance while placing local anesthesia around a nerve bundle you should:

Push through the resistance

**Pull needle back and abandon the block**

Pull needle back, reposition and then continue the block

Advance needle 0.5cm farther and push 1 mL of anesthesia
